# Resveratrol controlled the fate of porcine pancreatic stem cells through the Wnt/β-catenin signaling pathway mediated by Sirt1

**DOI:** 10.1371/journal.pone.0187159

**Published:** 2017-10-26

**Authors:** Shuanshuan Xu, Fen Sun, Lipeng Ren, Hong Yang, Na Tian, Sha Peng

**Affiliations:** College of Veterinary Medicine, Shaanxi Centre of Stem Cells Engineering & Technology, Northwest A&F University, Yangling, Shaanxi, P. R., China; Nankai University, CHINA

## Abstract

Porcine pancreatic stem cells (PSCs) are considered promising transplant materials that may be used to treat diabetes, but some problems, such as insufficient cell number and low differentiation efficiency, should be solved before its clinical application. Resveratrol is a natural polyphenolic compound that can alleviate the complications of diabetes. In this study, we aimed to explore the specific effect of resveratrol on porcine PSCs. We treated porcine PSCs with 10 μM, 25 μM resveratrol to explore the effect of resveratrol on porcine PSCs. We found that 10 μM resveratrol improved the proliferation of porcine PSCs, increased the expression of A-β-catenin (active β-catenin), *Pcna*, *C-Myc*, *Bcl-2* and sirtuin-1 (*Sirt1*), and decreased the expression of *P53*, *Caspase3*. While 25 μM resveratrol had almost opposite effect compared with 10 μM resveratrol group. The utilization of Dickkopf-related protein 1 (DKK1, Wnt signaling pathway inhibitor) and nicotinamide (*Sirt1* inhibitor) suggested that resveratrol regulated cell proliferation by controlling Wnt signaling pathway and this effect was mediated by *Sirt1*. Our results further revealed that 10 μM resveratrol promoted the formation of β-like cells regulated by Wnt/β-catenin signal pathway. Relatively low-dose resveratrol could improve porcine PSCs fate. It lays theoretical foundation for diabetes treatment with cell transplantation in future.

## Introduction

Diabetes has emerged as a crucial and serious health problem that requires prompt treatment [1]. In 2015, data revealed that approximately 415 million individuals suffered from diabetes worldwide, and about 642 million patients will be affected by diabetes in 2040 [1]. Two types of diabetes have been described. Type 1 diabetes is caused by insulin deficiency. Under this condition, functional β cells in the pancreas of patients are attacked by their immune system. To restore the insulin-secreting system that can respond effectively to multiple neural and hormonal signals, researchers proposed the transplantation of β cells derived from PSCs as a potential approach [2]. PSCs can also effectively cure type 2 diabetes, which is triggered by insulin resistance and impaired β cells [3]. However, the ability of the human pancreas to produce PSCs is inadequate. As such, studies on the applications of PSCs in diabetes therapy have been rarely performed.

With a high degree of similarity in structural and physiological functions between porcine and human, porcine PSCs are considered promising alternative therapies for diabetes [4–6]. However, PSCs have yet to satisfy medical quantification. To overcome this problem, we should reveal the mechanism of PSC proliferation and differentiation before its clinical application.

Resveratrol is a chemical constituent extracted from peanuts, grapes, and red wine [7–9]. This chemical exhibits oxidation and inflammatory resistance and plays a role in the treatment of cancer and inflammatory disease, particularly diabetic complications [10]. Resveratrol increases not only the life expectancy of yeast, worms, and flies but also the health span of rodents [11, 12]. Resveratrol also relieves progeroid traits in laminopathy-based progeria and rescues the decline in adult stem cells, which depends on NAD-dependent deacetylase *Sirt1* [13]. Resveratrol can alleviate H_2_O_2_-induced oxidative pressure of embryonic neural stem cells and can function as an activator of Sirt1, which is a NAD^+^-dependent protein deacetylase [14]. *Sirt1* is the closest homolog of Saccharomyces cerevisiae silent information regulator 2 and can regulate various metabolic pathways, such as IKK-β/IκВ/NF-κВ, AMPK, PI3K, and Wnt/β-catenin signaling pathways [15, 16]. A typical Wnt/β-catenin signaling pathway controls some biological events, such as proliferation, differentiation, development, and maintenance of stem cells [4, 17]. In an activated canonical Wnt signaling pathway, β-catenin accumulates in the cytoplasm, followed by β-catenin into the nucleus, Tcf/Lef binding, and then activate the downstream target genes [17]. Studies in breast tumor cells had found that Sirt1 and Wnt/β-catenin have a certain relationship [18]. In the present study, we aim to determine the effects of resveratrol on the proliferation and differentiation of porcine PSCs.

## Materials and methods

### Cell culture

The porcine PSCs line used in this study were established and kept by our group [4]. 0.125% (w/v) trypsin was utilized to digest the cells (Invitrogen, Carlsbad, CA, USA). The cells were cultured in Low glucose-DMEM (Invitrogen), containing 0.1 mM β-mercaptoethanol (Sigma), 10% FBS (fetal bovine serum), 1% Non-essential amino acid, 2 mM glutamine (Invitrogen). We replaced the culture medium each 24 h.

### Immunofluorescene staining

The immunofluorescene staining assays were performed as previously described [4]. The brief procedure was as follows: cells was fixed with 4% paraformaldehyde (PFA) for 12 min, and rinsed by phosphate buffer solution (PBS, pH = 7.4) for three times. Then, cells were treated with 0.1% Triton X-100 for 10 min. After three washes with PBS, the cells was blocked with 1% bovine serum albumin (BSA) at 37°C for 1 h. Afterwards, we treated the cells with anti-P53 (1:200, Rabbit IgG, Cell Signaling Technology) for 12 h at 4°C, then washed three times with PBS and treated with the corresponding secondary antibody (1:500, Goat anti-Rabbit IgG, ZSGB-BIO) for 1 h at 37°C. In the end, we used the Hoechst33342 (Sigma) to stain the nuclei for 5 min at room temperature. We used the Leica fluorescent microscope to capture and analyze the images.

### BrdU assay

Procedure for treating cells was as follows: the concentration of BrdU (Sigma, St Louis, MO, USA) was 30 mg/ml. We treated the cells with BrdU for 6 h at RT (room temperature) and fixed them in methanol and acetone solution (1:1) for 10 min. After three washes with PBS, 2 M HCl was used to denature cells for 45 min at RT. Then the cells were neutralized with 0.1 M sodium borate at RT for 15 min. The treatment procedure of primary (Mouse anti-BrdU IgG1, BOSTER, 1:100) and secondary antibodies (FITC-labeled goat anti-mouse IgG, ZSGB-BIO, 1:500) was as described in immunofluorescene staining assays. Image J software was used to count the number of BrdU positive cells. In order to ensure the reliability of this experiment, we repeated the assay three times, and 3 fields were randomly chosen for statistical analysis each time.

### CCK-8 assay

We seeded porcine PSCs on 96-well plate. Then we treated the cells with resveratrol for 24 h. Each group was performed in six wells. In the end, we used the CCK-8 (Beyotime) solution to treat the cells for 1 h at 37°C. We used the microplate reader to measure the results.

### qRT-PCR

We used the qRT-PCR (quantitative reverse transcriptase-polymerase chain reaction) to detect the genes expression. qRT-PCR was performed with SYBR @ PremixExTaq^TM^ (TaKaRa, Biotech. Co. Ltd) and was made up 15 μL reaction system containing 7.5 μL SYBR @ PremixExTaq^TM^, 0.3 μL sense primer, 0.3 μL antisense primer, 5.7 μL distilled water, 1.2 μL template. Reaction conditions were as follows: 94°C for 2 min, followed by 40 cycles at 94°C for 10 s, 60°C for 15 s, 72°C for 30 s. And a melting curve was programmed. We used the β-actin as a reference. The primer sequences for qRT-PCR were shown in Table 1.

**Table 1:** The sequence of primers used for qRT-PCR.

Primers	Forward	Reverse
C-Myc	5’- ctggtgggcgagatcatca-3’	5’-cactgccatgaatgatgttcc3’
Pcna	5’-agtggagaacttggaaatggaa-3’	5’-agtggagaacttggaaatggaa-3’
P53	5’-tttccgtctagggttcctg-3’	5’-cgtcatgtgtccaacttc-3’
Bcl-2	5’-atgtgtggagagcgtcaa-3’	5’-ctagggccatacagctccac-3’
Caspase3	5’-cagacagtggtgctgaggatga-3’	5’-gctacctttcggttaacccga-3’
Sirt1	5’-cgagaattcgttgaaagatggcgg-3’	5’-gcgcggatcccattcaatttgacat-3’
β-actin	5’-gcggcatccacgaaactac-3’	5’-tgatctccttctgcatcctgtc-3’
insulin	5’-aagcgtggcatcgtggag-3’	5’-tcaggactttattgggtttgg-3’
Glut-2	5’-ttgccttggatgagttatgtga-3’	5’-gcgtggtccttgactgaaaa-3’
NeuroD1	5’- tcttgcgttcaggcaaaagc-3’	5’-aagtccgaggattgagctgc-3’
CyclinD1	5’-gtgaaaaagagccgcctgc-3’	5’-cggatggagttgtcggtgtag-3’
MafA	5’-ttcagcaaggaggaggtcat-3’	5’-acaggtcccgctctttgg-3’

### Western blotting analysis

The protein was separated by 1×sodium dodecyl sulfate polyacrylamide gel electrophoresis (SDS-PAGE). We transferred the protein to 0.22 μm PVDF membrane by using wet transfer. After being blocked in 8% non-fat milk overnight, the membrane were incubated by primary antibodies including anti-Pcna (1:1000, Mouse IgG, Cell Signaling Technology), anti-C-Myc (1:1000, Rabbit IgG, Cell Signaling Technology), anti-P53 (1:500, Rabbit IgG, Cell Signaling Technology), anti-caspase3 (1:500, Rabbit IgG, Beijing Biosynthesis Biotechnology) and anti-β-tublin (1:1000, Mouse IgG, Sino Biological), anti-Sirt1 (1:500, Rabbit IgG, Sangon Biotech), anti-A-β-catenin (1:1000, Rabbit IgG, Abcam), anti-PDX1 (1:1000, Rabbit IgG, Sangon Biotech), anti-β-catenin (1:500, Mouse IgG, Santa Cruz), anti-Acetyl-β-catenin (lys49) (1:1000, Rabbit IgG, Cell Signaling Technology), anti-GAPDH (1:1000, Mouse IgG, Tianjin Sungene Biotech) at 4°C for 12 h. Horse-radish peroxidase-conjugated anti-rabbit and anti-mouse (1:1000, Beyotime) treated the membrane at 37°C for 1 h. The blots were viewed and analyzed by Tanon-4200 (Shanghai Tianneng Corporation, China).

### Cell apoptosis assay

We used apoptosis detection kit (MultiSciences Biotech, Hangzhou, China) to detect cell apoptosis. Cell apoptosis distribution was stained by Annexin V-FITC and PI. The detail of the procedure was described in our previous study [19]. Before analysis, we washed and resuspended cells by PBS. Then the results were assessed and analyzed by flow cytometry.

### Construction of Sirt1 overexpression vector

We designed Sirt1 primers and cloned it according to the CDS region of the Sirt1 gene (NM_001145750.2) of porcine. The primer sequence as follows:

Farward-5’- GGAATTCCAGAGGCAGTTGAAAGATGGCG-3’;

Reverse-5’- CGGATCCGCCCTAATGCTGGTGGAACAAT-3’.

Sirt1 was cloned from the cDNA of porcine PSCs. The fragment was then ligated with the linear vector pCDH-CMV-MCS-EF1 (Vector). The constructed successfully vector was named as pCDH-CMV-MCS-EF1-Sirt1 (Ov-Sirt1). Then we used Ov-Sirt1 to transfect the 293T for lentivirus generation. The 293T cells were panted at a 90% density before transfection. Added the transfection regent (Turbofect, Thermo Fisher Scientific Inc., Shanghai, China), Ov-Sirt1 or pCDH-CMV-MCS-EF1, PAX2, VAVG into the Opti-MEM (Invitrogen, Shanghai, China), incubated for 12 h. Then, the medium was replaced by the Virus packaging solution (2% FBS, 0.1mM BME, 2mM L-glutamin, 1% non-essential amino acid and CD Liquid). The medium contained lentivirus was collected after 60 h. Porcine PSCs were seeded in a 6-well at a density of 5×10^5^. The cells were transfected by lentivirus containing 10 μg/ml polybrene. After 12 h, we replaced the medium with fresh low glucose-DMEM.

### Induction of porcine PSCs into β-like cells

We used two steps protocol to get the β-like cells. The RPMI H-DMEM (Gibco) was used as base medium, added 10% FBS (Gibco), 2 mM glutamine (Invitrogen), 1 mM β-mercaptoethanol (Sigma), 10 mM nicotinamide (Sigma), 0.2% B27 (STEMCELL Technologies, Inc.), 1 mM sodium pyruvate (Invitrogen), 0.2% Insulin Transferrin Selenium (Sigma) and 25 ng/mL Activin A (Sigma) as the first stage induction medium. After 3 days, we added 25 mM zinc acetate, 10 nM exendin 4 (Sigma) and 4 nM beta-cellulin (Sigma) to the first stage induction medium as second stage induction medium. Then, we seeded and cultured the cells to ultra-low attachment plates for another 7 days. The induction medium was changed every 2 days.

### Dithizone (DTZ) staining

We seeded suspension-induced cell masses in 96-well plate (n = 5). After attaching, the masses were fixed with 4% PFA for 13 min at RT. Then, they were stained with freshly prepared DTZ (Sigma) for 15 min at RT. The masses were photographed by an optical microscope after three washes.

### Statistical analysis

We repeated each experiment three times. The data are presented as mean ± SDs, and the difference was analyzed by two-tailed independent-sample Student t test. We considered statistically significant when P values < 0.05. *p < 0.05, **p < 0.01.

## Results

### Resveratrol elicited different effects on porcine PSCs proliferation at different concentrations

**Fig 1:**
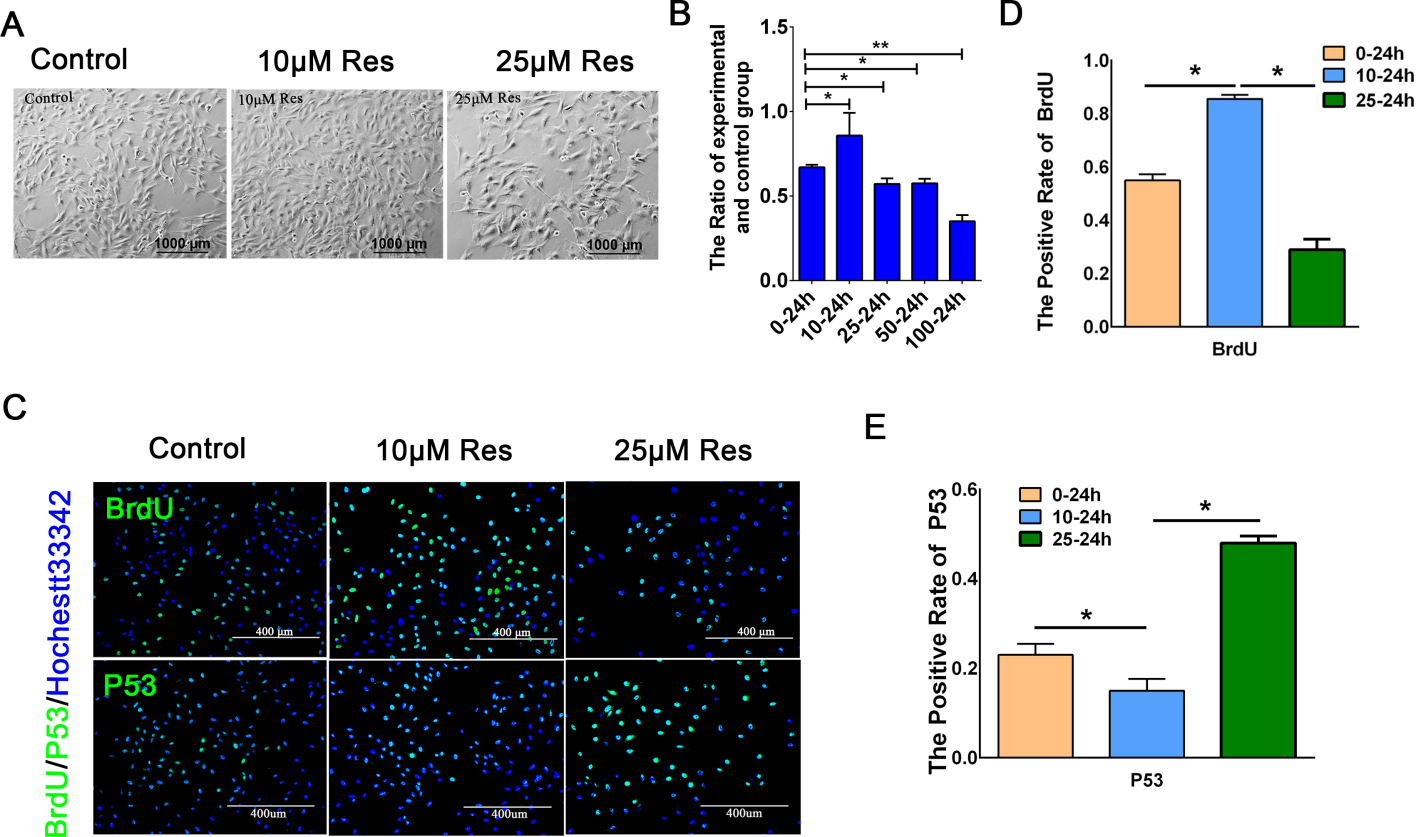
Resveratrol affected porcine PSC proliferation dependent on treatment concentration. (A) The morphology of porcine PSCs treated with 1‰ DMSO (control), 10 μM resveratrol (Res), and 25 μM Res for 24 h. Bar = 1,000 μm. (B) Results of the CCK-8 assay of porcine PSCs treated with different concentrations of resveratrol (0, 10, 25, 50, and 100 μM) for 24 h. (C) Representative images of BrdU staining and immunofluorescence staining of the apoptosis marker P53. Bar = 400 μm. (D) Quantification of BrdU-positive porcine PSCs. E, Quantification of P53-positive porcine PSCs. *p < 0.05, **p < 0.01.

A previous study reported that resveratrol could inhibit MCF-7 cell growth in a time-dependent and concentration-dependent manner and induce its apoptosis by activating the p38–p53 signaling pathway [20]. Our study examined the effects of resveratrol on porcine PSCs proliferation under different concentrations. CCK-8 assay demonstrated that resveratrol could enhance porcine PSCs proliferation at a low dose (10 μM), whereas it inhibited this effect at a higher dose (>10 μM) (Fig 1B). The cell density increased obviously after treatment with 10 μM resveratrol compared with the control group (0 μM). However, it decreased when the cells were treated with 25 μM resveratrol (Fig 1A). The result of the growth curve showed that 10 μM resveratrol can improve the viability of porcine PSCs compared with two other groups, while 25 μM have a completely opposite effect (S1C Fig). Further, BrdU-positive cells were significantly increased in the 10 μM resveratrol treatment group compared with the control group, but obviously decreased in 25 μM resveratrol group compared with the control and 10 μM resveratrol groups (Fig 1C and 1D). And P53-positive cells in 10 μM treatment group were less than that of the control group, but it was higher than that of two other groups when porcine PSCs were treated with 25 μM resveratrol (Fig 1C and 1E). As shown in S1A and S1B Figs, PDX1 expression did not been observed after resveratrol treatment (S1A–S1B Fig). These results indicated that porcine PSCs proliferation could be regulated by resveratrol and different treatment concentrations could lead to opposite influences without changing their characteristics.

### Resveratrol regulated porcine PSCs proliferation through the Wnt/β-catenin signaling pathway

**Fig 2:**
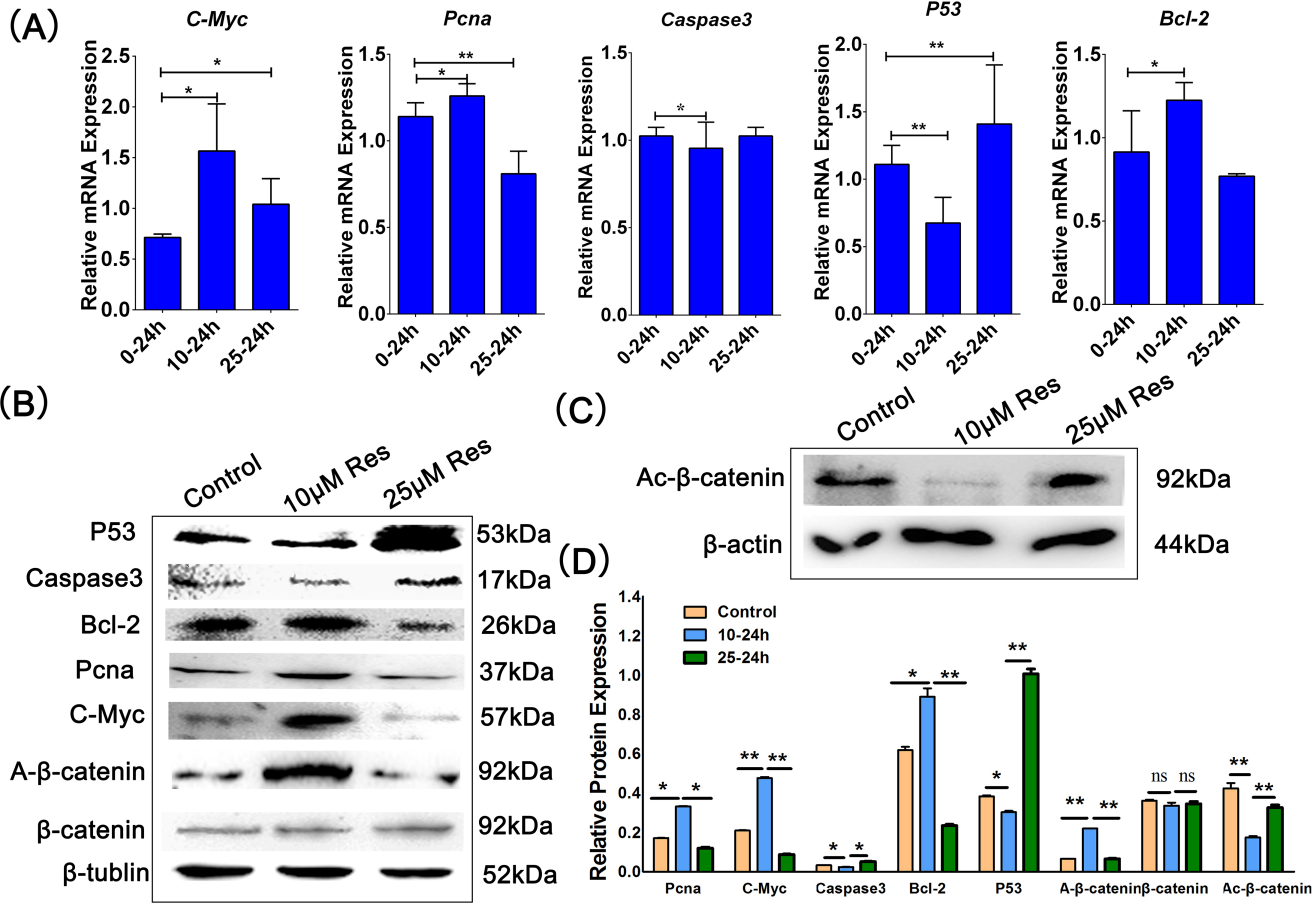
Resveratrol regulated porcine PSC proliferation through the Wnt/β-catenin signaling pathway. (A) Porcine PSCs were treated with 1‰ DMSO (control), 10 μM Res, and 25 μM Res for 24 h. The expression of proliferation-related markers, including *C-Myc*, *Pcna*, *Caspase3*, *P53*, and *Bcl-2* in porcine PSCs detected by qRT-PCR. (B)—(D) Western blotting and the corresponding quantification results of Pcna, C-Myc, P53, Caspase3, Bcl-2, A-β-catenin, Ac-β-catenin and β-catenin expression. *p < 0.05, **p < 0.01.

**Fig 3:**
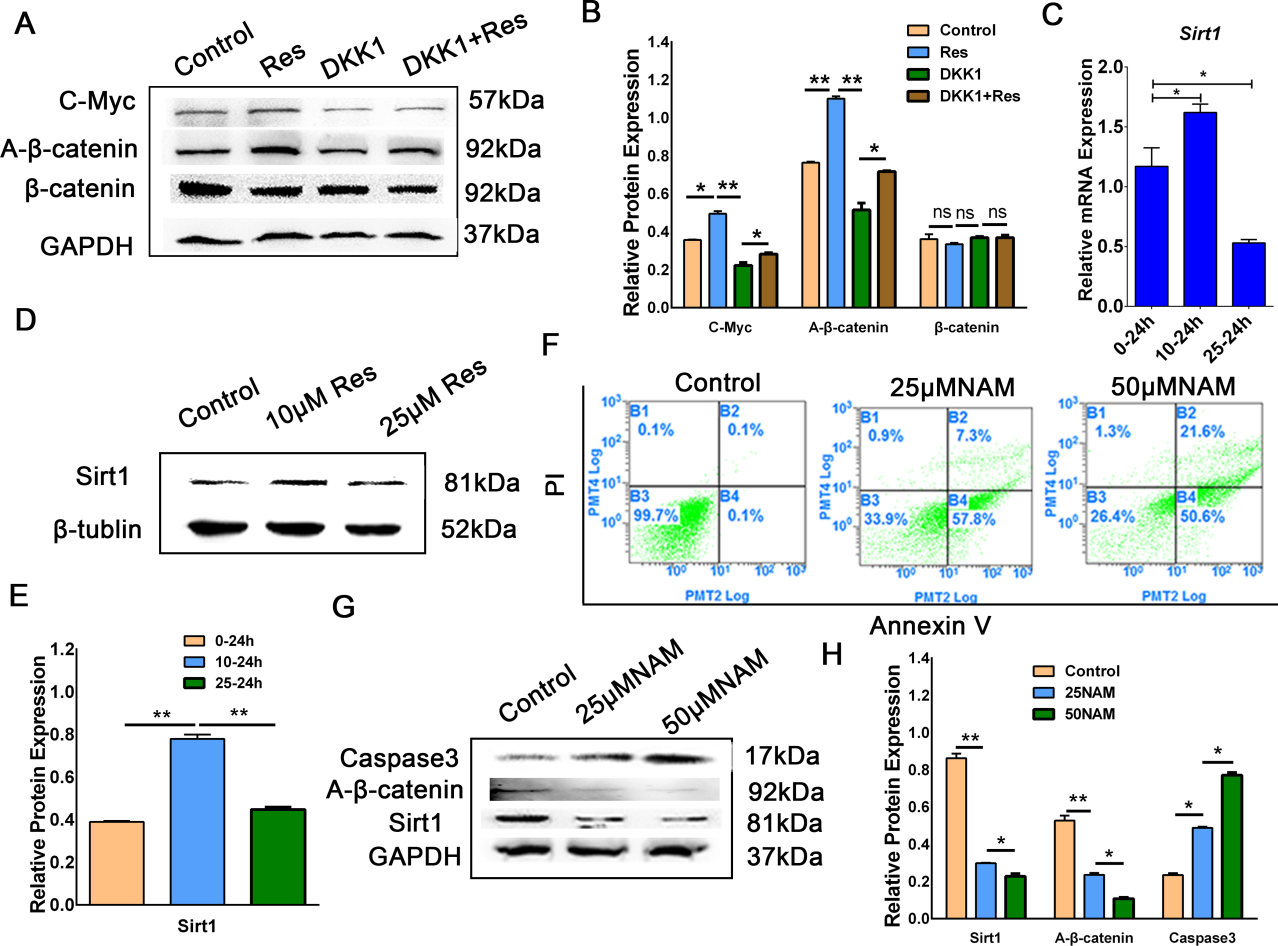
Resveratrol activated the Wnt/β-catenin signaling pathway mediated by Sirt1. (A) Porcine PSCs were treated with 1‰ DMSO (control), 10 μM Res, 250 ng/mL DKK1, and 10 μM Res + 250 ng/mL DKK1 for 36 h. Western blotting results of C-Myc, A-β-catenin, and β-catenin expression. (B) Corresponding quantification of the Western blotting results shown in (A). (C) The mRNA expression of Sirt1 in porcine PSCs after treatment with resveratrol (control, 10 μM, and 25 μM) analyzed by qRT-PCR. (D)—(E) The protein expression of Sirt1 in porcine PSCs after treatment with resveratrol detected by Western blotting and the corresponding quantification results of Western blotting. (F) Porcine PSCs were treated with 1‰ PBS (control), 25 μM NAM, and 50 μM NAM for 36 h. Apoptosis of the cells was detected by flow cytometry. (G)—(H) Western blotting and the corresponding quantification results of Sirt1 and Caspase3 expression. *p < 0.05, **p < 0.01.

We detected the genes associated with proliferation and apoptosis, like *C-Myc*, *Pcna*, *Caspase3*, *P53*, and *Bcl-2*, to explore the molecular mechanism of resveratrol in regulating porcine PSCs proliferation. The results showed that the expression levels of *Pcna* and *Bcl-2* were obviously upregulated at the mRNA and protein levels when porcine PSCs were treated with 10 μM resveratrol, while they were downregulated in 25 μM treatment groups compared to two other groups (Fig 2A–2D). Moreover, 10 μM resveratrol caused the downregulation of *P53* and *Caspase3* expression, whereas 25 μM resveratrol had completely opposite roles at the mRNA and protein levels (Fig 2A–2D). Although the expression of *C-Myc* had not been downregulated at the mRNA level compared with the control group, it was significantly downregulated at the protein level after treatment with 25 μM resveratrol (Fig 2A–2D). Wnt signaling pathway, which can mediated the expression of *Pcna* and *C-Myc*, is a key regulator of proliferation and differentiation in many stem cells [17]. Thus, we also detected the expression of A-β-catenin, which is a crucial molecule of the Wnt signal pathway in this study. As shown in Fig 2B and 2D, 10 μM resveratrol upregulated the expression of A-β-catenin in porcine PSCs, even though its expression was almost unchanged in 25 μM resveratrol group (Fig 2B and 2D). The expression of Acetyl-β-catenin was inhibited by 10 μM resveratrol (Fig 2C). Then, we used the DKK1, which was an inhibitor of Wnt signaling pathway, to determine the function of the Wnt/β-catenin during porcine PSCs proliferation. We found that 10 μM resveratrol could remit the inhibitory influence of DKK1 on the expression of *C-Myc* and A-β-catenin (Fig 3A and 3B). These results indicated that resveratrol could affect porcine PSCs proliferation by regulating Wnt/β-catenin signal pathway.

### Resveratrol activated the Wnt/β-catenin signaling pathway mediated by Sirt1

**Fig 4:**
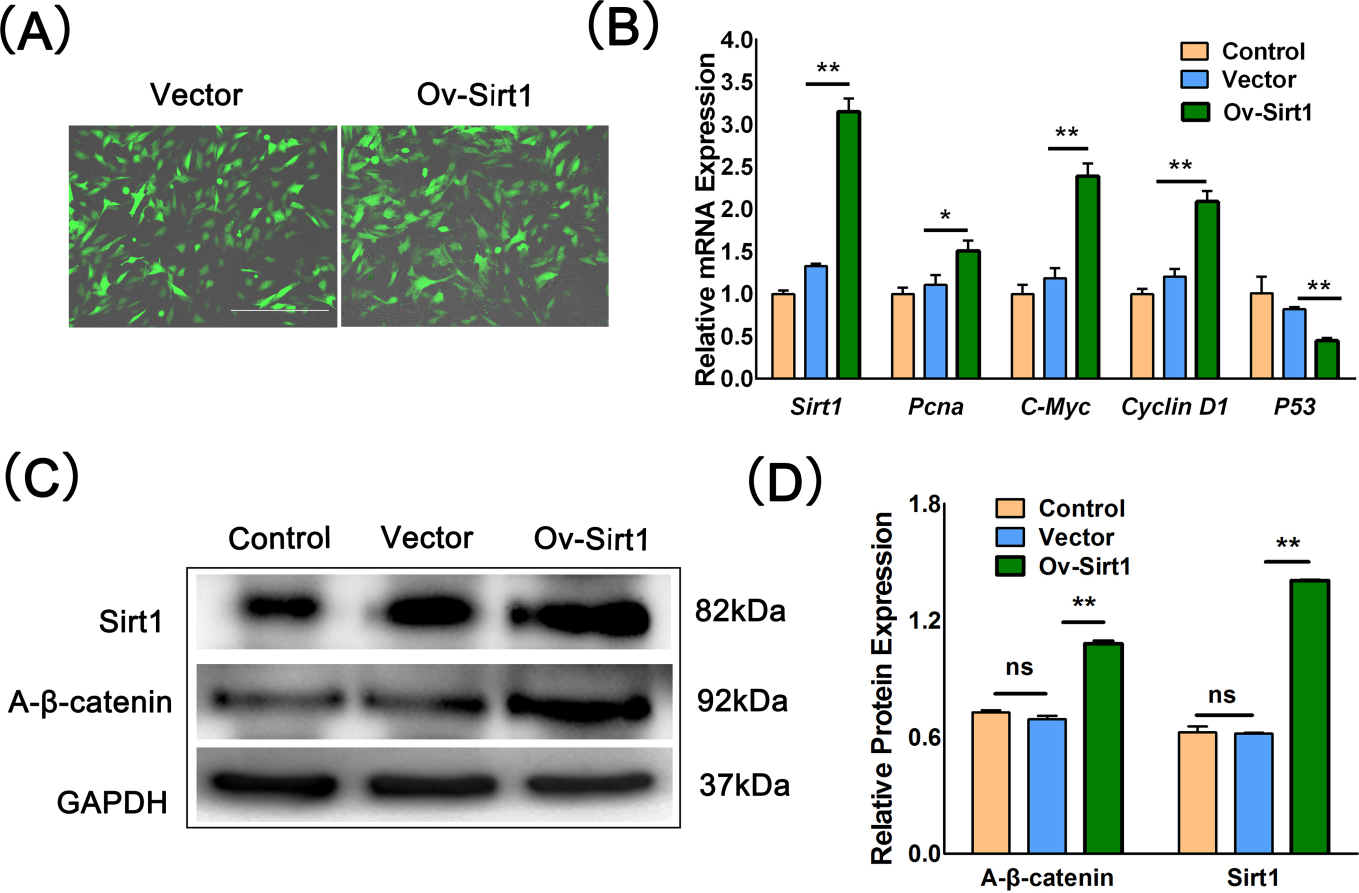
Sirt1 promoted the proliferation of porcine PSC. (A) Pictures of the cells transfected with Vector (pCDH) or Ov-Sirt1. Bar = 400μm. (B) The relative mRNA expression of *Sirt1*, *Pcna*, *C-Myc*, *CyclinD1* and *P53*. (C)–(D) Western blotting and the corresponding quantification results of Sirt1 and A-β-catenin expression. *p < 0.05, **p < 0.01.

Resveratrol can activate the expression of *Sirt1* which benefit the survival of human mesenchymal stem cells [21]. To investigate whether resveratrol had the similar effects in porcine PSCs, the *Sirt1* expression was examined after treatment with different concentration of resveratrol. Interestingly, although the expression of *Sirt1* was obviously upregulated in the 10 μM resveratrol group (Fig 3C–3E), it was downregulated at the mRNA level and almost unchanged at the protein levels in the 25 μM resveratrol group compared with control group (Fig 3C–3E). Nicotinamide (NAM), which is a frequently used inhibitor of *Sirt1*, was utilized to further clarify the effect of *Sirt1* on porcine PSCs. We found that NAM could significantly enhance the apoptosis rate of porcine PSCs and the expression of *Caspase3* in the 25 and 50 μM groups (Fig 3F–3H). Moreover, the expression of A-β-catenin was decreased as *Sirt1* inhibited. And 10 uM resveratrol inhibited the acetylation of beta-catenin. When the cells were transfected Ov-Sirt1, we found that A-β-catenin expression subsequently increased (Fig 4C and 4D). And the expression level of *Pcna*, *C-Myc* and *CyclinD1* in Ov-Sirt1 group was higher than the empty vector group (Fig 4B). These results indicated that 10 μM resveratrol could increase the expression of Sirt1 which activated the Wnt/β-catenin signaling pathway subsequently.

### Resveratrol supported the formation of β-like cells in the high-glucose environment

**Fig 5:**
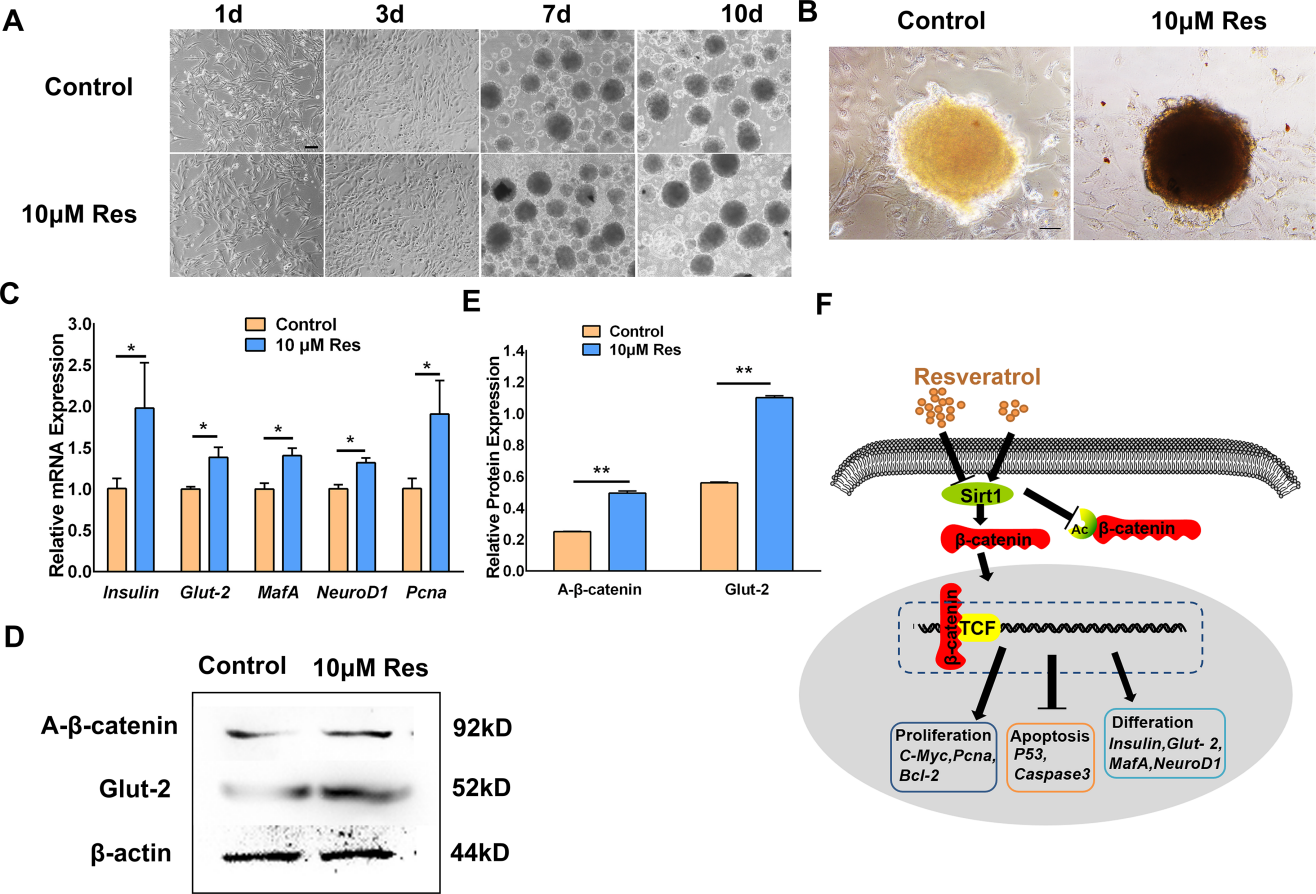
Resveratrol supported the formation of β-like cells in the high-glucose environment. (A) After adding 10 μM resveratrol, the cell shape was observed at 1, 3, 7, and 10 days in high-glucose induction system. Bar = 100 μm. (B) DTZ staining. Bar = 100 μm. (C) The qRT-PCR results of *insulin*, *Glut-2*, *MafA*, *NeuroD1*, and *Pcna*. (D)—(E) Western blotting and the corresponding quantification results of A-β-catenin and Glut-2 expression. (F) Diagram illustrating the mechanism of resveratrol regulating porcine PSC proliferation and differentiation. *p < 0.05, **p < 0.01.

10 μM resveratrol was added to traditional high-glucose induction system [22]. The results showed that the edges of β-like cell masses were more regular, and reddish brown was deeper in the resveratrol group than that of control group detected by DTZ staining (Fig 5A and 5B). Moreover, 10 μM resveratrol promoted the expression of *Pcna* in β-like cell masses compared with control (Fig 5C). The results of qRT-PCR indicated that the expression levels of *insulin*, *Glut-2*, *MafA*, *and NeuroD1*, which are related to the maturity of β cells, were also upregulated in the resveratrol groups (Fig 5C). And 10 μM resveratrol increased the protein expression of A-β-catenin and Glut-2 (Fig 5D and 5E). These results indicated that 10 μM resveratrol improved the state of porcine PSCs and promoted the formation of β-like cells. Moreover, this effect may be caused by activation of Wnt signal pathway.

## Discussion

The proliferation and differentiation mechanism of porcine PSCs, which are optimum alternatives to human PSCs during transplantation therapy for diabetes, should be investigated. This study revealed that resveratrol could regulate porcine PSCs proliferation and differentiation into β-like cells through the Wnt/β-catenin signaling pathway.

In our study, resveratrol could affect the fate of porcine PSCs in a concentration-dependent manner. In particular, 10 μM resveratrol could promote porcine PSCs proliferation and inhibit its apoptosis, whereas 25 μM resveratrol elicited a completely opposite effect. *C-Myc* and *Pcna* are associated with DNA synthesis and implicated in cell proliferation [23, 24]. All of these three genes, namely *P53*, *Casepase3* and *Bcl-2*, are closely associated with cell apoptosis and can act as markers to evaluate apoptosis [25–27]. *Pcna*, *C-Myc*, *P53*, *Caspase3*, and *Bcl-2* act as downstream genes of the Wnt/β-catenin pathway, which plays a pivotal function during many biological processes, such as cell proliferation, differentiation, development, and maintenance of stem cells [4, 17]. The accumulation of β-catenin in the nucleus is the key to the function of Wnt signaling pathway [28]. When β-catenin is phosphorylated, the phosphorylated β-catenin binds to APC (adenomatous polyposis coli) and is subsequently degraded [29]. A-β-catenin did not undergo phosphorylation. It binds to Tcf into a complex and activates the target gene of the Wnt signaling pathway. Expression of *C-Myc* was activated by A-β-catenin, the effect was mediated through Tcf binding sites in the *C-Myc* promoter [30]. Wnt/β-catenin signaling pathway interacts with the P53 signaling pathway to regulate cell proliferation and apoptosis [31]. And the key protein Tcf-4 of the Wnt signaling pathway regulates the transcription of P53 [32]. Bcl-2 family could inhibit the release of cytochrome c which could active Caspae3 [33]. In Figs 1 and 2, the expression levels of *Pcna*, *C-Myc*, *Bcl-2*, and A-β-catenin were upregulated in porcine PSCs after treatment with 10 μM resveratrol, whereas the expression levels of *P53* and *Caspase3* were downregulated. Moreover, the effects of resveratrol are reversed by DKK1, which is the inhibitor of the Wnt/β-catenin signaling pathway. These results illustrated that the effect of resveratrol on porcine PSC proliferation was regulated by the Wnt/β-catenin signaling pathway.

Resveratrol can protect cultured H9C2 cardiac cell line cells by activating *Sirt1* [34]. Our findings showed that 10 μM resveratrol could upregulate the expression of *Sirt1* in porcine PSCs. However, *Sirt1* was downregulated at the mRNA level and was almost unchanged at the protein levels in the 25 μM resveratrol group compared with the two other groups (Fig 3). These results indicated that the effect of resveratrol on *Sirt1* activation is dependent on the treatment concentration. When treated with high concentrations of resveratrol, it might cause cell physiological activity disorders, such as endoplasmic reticulum stress, at this time, Sirt1 may not be the main factor in regulating cell physiological activity [35]. And the Sirt1 expression of cells treated with high-concentration would not increase more than the low-concentration treatment.Sirt1 can activate the Wnt/β-catenin signaling pathway and increase A-β-catenin translation into the nucleus [34]. The cross-talk between the Wnt/β-catenin signaling pathway and the sirtuin/Foxo1 longevity pathway can provide protection against muscular pathology [36].Sirt1 mediates the epigenetic silence of these genes by degrading the histones of the Wnt extracellular *sFRP-1* and *sFRP-2* motilin regions [37, 38]. This means that Sirtl can up-regulate the Wnt signaling by inhibiting extracellular antagonists of the Wnt signaling pathway. In addition, Sirt1 binds to all subtypes of the Dishevelled protein family in a number of different cell lines, such as human embryonic kidney cell lines, colon cancer cell lines, and breast cancer cell lines. Activation of Sirt1 promotes the expression of the Wnt/Dvl target gene by increasing the expression of the Dishevelled protein, whereas the inhibition of Sirt1 reduces the abundance of Dishevelled protein expression, and Dacts can bind directly to Dishevelled [39]. In our study, we found that once *Sirt1* was inhibited by NAM, the expression of A-β-catenin was also reduced (Fig 3G and 3H). Thus, we deduced that a cross-talk occur between Wnt/β-catenin and Sirt1 after resveratrol treatment in porcine PSCs. As we all know, Sirt1 is a deacetylase. It regulates Wnt signal pathway by increasing the deacetylation of β-catenin to promote MSCs (Mesenehymal stem cell) proliferation and suppresses the adipogenesis [40]. We found that the acetylation level of β-catenin was reduced when the cells treated with 10 uM resveratrol (Fig 2C). So 10 μM resveratrol reduced the acetylation degradation of β-catenin, and increased the nuclei accumulation of A-β-catenin.

Some studies have shown that resveratrol promotes the differentiation of marrow stroma cell (MSC) to osteoblast, and this process is adjusted by the Wnt/β-catenin signaling pathway [41]. Furthermore, resveratrol promotes the mouse embryonic stem cells differentiation to cardiomyocytes and shows no toxicity on cell viability [42]. Resveratrol elicits dosage-dependent effect on the hUC-MSCs’ self-renewal and neural differentiation [43]. In present study, we found that 10 μM resveratrol promoted the formation of β-like cells accompanied with upregulation of A-β-catenin expression (Fig 5). These observations suggested that resveratrol may participate in porcine PSCs differentiation toward to β-like cells by activating Wnt/β-catenin signaling pathway. Next, further studies should focus on the concrete mechanism of resveratrol on the differentiation of porcine PSCs.

In conclusion, in this study, we revealed the roles of resveratrol on the fate of porcine PSCs. It was closely related to treatment concentration of resveratrol. We concluded the possible mechanism during this process (Fig 5F). A lower concentration of resveratrol could activate Wnt/β-catenin signal pathway possibly mediated by Sirt1, then induce transcriptional activation of downstream target genes associated with proliferation, apoptosis and differentiation molecules. On the contrary, a higher concentration of resveratrol inhibited porcine PSCs proliferation and promoted its apoptosis through the opposite mechanism. This study revealed that a relatively low concentration of resveratrol could promote porcine PSCs proliferation and differentiation through activating Wnt/β-catenin signaling pathway mediated by *Sirt1*.

## Supporting information

S1 Fig Resveratrol did not alter the characteristics of porcine PSCs. (A) Immunofluorescene staining of the PDX1 in porcine PSCs. Bar-400μm. (B) Western blotting of PDX1. (C) The growth curve of porcine PSCs treated with resveratrol (control, 10 μM, 25 μM) for 24 h.
(TIF)

S1 File The original uncropped and unadjusted pictures of western blot in Figs 2, 3, 4, 5 and S1. (RAR)
