# Supplementary figures and images for: Resveratrol controlled the fate of porcine pancreatic stem cells through the Wnt/β-catenin signaling pathway mediated by Sirt1
Source: PLoS One. 2017 Oct 26;12(10):e0187159. doi: 10.1371/journal.pone.0187159 (PMC5658170; doi:10.1371/journal.pone.0187159)

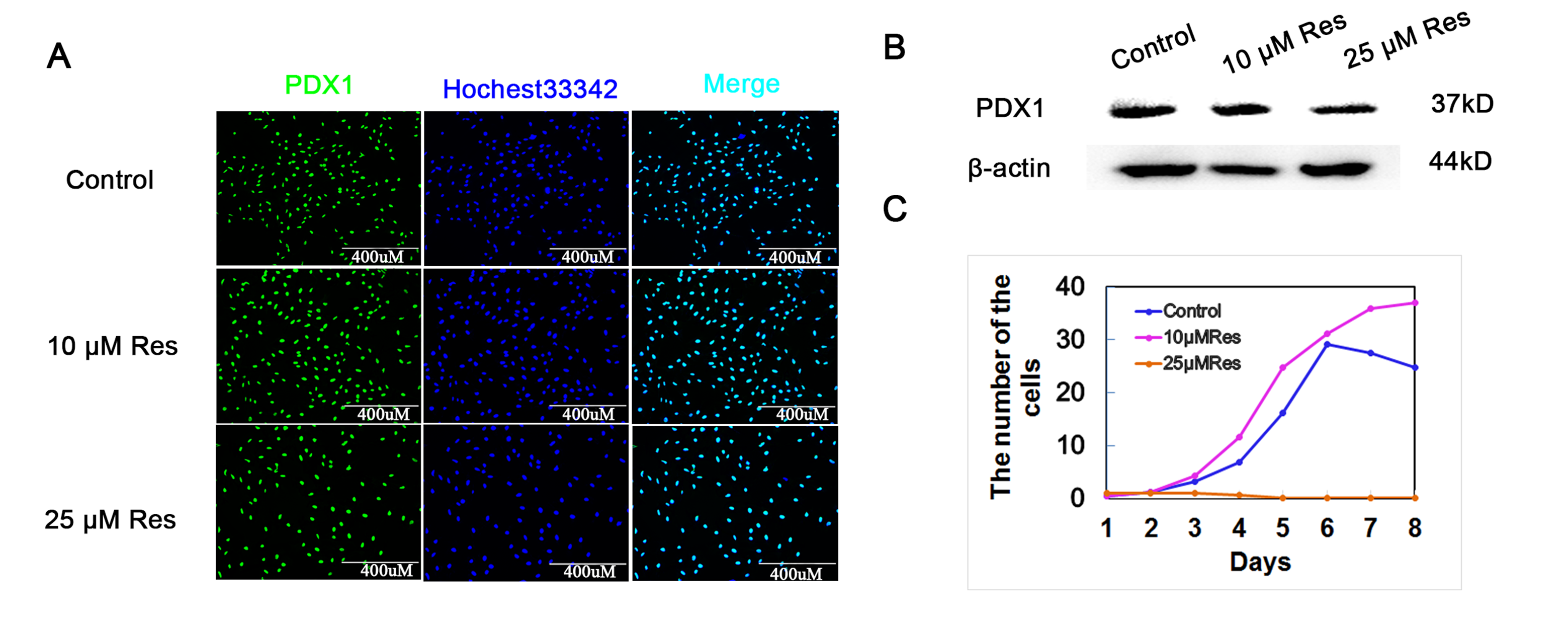

Supplement: S1 Fig — (A) Immunofluorescene staining of the PDX1 in porcine PSCs. Bar-400μm. (B) Western blotting of PDX1. (C) The growth curve of porcine PSCs treated with resveratrol (control, 10 μM, 25 μM) for 24 h. (TIF) [file pone.0187159.s001.tif]
